# Insecticide resistance in *Anopheles gambiae* from the northern Democratic Republic of Congo, with extreme knockdown resistance (*kdr*) mutation frequencies revealed by a new diagnostic assay

**DOI:** 10.1186/s12936-018-2561-5

**Published:** 2018-11-06

**Authors:** Amy Lynd, Ambrose Oruni, Arjen E. van’t Hof, John C. Morgan, Leon Bwazumo Naego, Dimitra Pipini, Kevin A. O’Kines, Thierry L. Bobanga, Martin J. Donnelly, David Weetman

**Affiliations:** 1 Liverpool School of Tropical Medicine, Liverpool, UK; 2 Avenue de l’infirmerie, Quartier Yola Bokonzo, Gemena, Sud Ubangi Democratic Republic of Congo; 3 University of Kinshasa, Kinshasa, Democratic Republic of Congo

## Abstract

**Background:**

Mutations in the voltage-gated sodium channel at codon 1014 confer knock-down resistance (*kdr*) to pyrethroids in a wide range of insects. *Anopheles gambiae* exhibits two mutant alleles at codon 1014, serine and phenylalanine; and both are now widespread across Africa. Existing screening methods only allow for one resistant allele to be detected per assay. A new locked nucleic acid (LNA) qPCR assay was developed for the simultaneous detection of both mutant alleles and the wild type allele in a single assay. This tri-allelic detection assay was assessed as part of a study of the insecticide resistance in *An. gambiae* sensu stricto (s.s.) in the previously un-sampled area of Nord Ubangi, Democratic Republic of the Congo.

**Methods:**

Samples from three sites were tested for insecticide susceptibility using WHO bioassays, with and without the synergist PBO preceding pyrethroid exposures, and were subsequently analysed for frequency and resistance-association of the V*gsc*-1014 and *Vgsc*-N1575Y mutations. Results from the LNA-*kdr* 1014 assay were compared to results from standard TaqMan-*kdr* assays.

**Results:**

*Anopheles gambiae* sensu lato (s.l.) was by far the predominant vector captured (84%), with only low frequencies of *Anopheles funestus* s.l. (9%) detected in Nord Ubangi. Molecular identification found *An. gambiae* s.s. to be the principal vector (99%) although *Anopheles coluzzii* was detected at very low frequency. *Anopheles gambiae* were susceptible to the carbamate insecticide bendiocarb, but resistant to DDT and to the pyrethroids permethrin and deltamethrin. Susceptibility to both pyrethroids was partially restored with prior exposure to PBO suggesting likely involvement of metabolic resistance. *Anopheles gambiae* s.s. was homozygous for *kdr* resistant alleles with both the L1014F and L1014S mutations present, and the N1575Y polymorphism was present at low frequency. The LNA-*kdr* assay simultaneously detected both resistant alleles and gave results entirely consistent with those from the two TaqMan-*kdr* assays.

**Conclusion:**

This study provides rare data on insecticide resistance and mechanisms in *Anopheles* from the centre of Africa, with the first detection of N1575Y. Nord Ubangi populations of *An. gambiae* s.s. show insecticide resistance mediated by both metabolic mechanisms and *Vgsc* mutations. The LNA-*kdr* assay is particularly suitable for use in populations in which both 1014S and 1014F *kdr* alleles co-occur and provides robust results, with higher throughput and at a quarter of the cost of TaqMan assays.

**Electronic supplementary material:**

The online version of this article (10.1186/s12936-018-2561-5) contains supplementary material, which is available to authorized users.

## Background

Malaria is a major cause of mortality and morbidity in The Democratic Republic of the Congo (DRC), with over 40,000 deaths per year [1]. It is estimated that 60% of the country’s population live in areas with an average *Plasmodium falciparum* prevalence above 50% (hyper-endemic to holoendemic transmission), making the DRC one of the countries with the most intense transmission [2]. Efforts to reduce the malaria burden are focusing on case management and treatment, and on vector control via the distribution of long-lasting insecticide-treated nets (LLINs) [2]. Successful implementation of a vector control programme is reliant on knowledge of vector species and their resistance to insecticides. Few recent studies have been published on the vector species of the DRC or their insecticide resistance and those that exist are principally concerned with locations to the South and East of the country. Over 60 species of *Anopheles* have been described in the DRC, with *Anopheles gambiae* sensu lato (s.l.) and *Anopheles funestus* thought to be the main malaria vectors, but other species, such as *Anopheles pharoensis, Anopheles moucheti*, and *Anopheles coustani* are potentially important for transmission (reviewed in [2]). In the *An. gambiae* s.l. species group, *An. gambiae* sensu stricto (s.s.) was found to be the predominant vector in eastern DRC, whereas *Anopheles coluzzii* was the main species found in Bandundu in the West. Both species were found in sympatry in several locations including Kinshasa, as well as Kisangani and Lodja (central West) and Kalemie (East). Only *An. gambiae* s.s. was found in Equateur Province (North West) [1, 3–5].

Contemporary data on insecticide resistance status in malaria vectors is improving but remains sparse for DRC and central Africa generally [6]. A study in 2009 of four locations, Kingasani and Kimpese in the South West, Bolenge, in the West, and Katana located in the East demonstrated that all *An. gambiae* s.s. populations were resistant to DDT, three were resistant to the pyrethroids deltamethrin, permethrin, lambda-cyhalothrin, and a single population was resistant to the organophosphate malathion [3]. In 2012, *An. gambiae* s.s. in the North East were found to be resistant to deltamethrin, DDT and bendiocarb. Pre-exposure to PBO (piperonyl butoxide), a synergist that inhibits the activity of cytochrome P450 enzymes and some esterases which may be involved in the detoxification of pyrethroids, significantly increased mortality in bioassays suggesting that metabolic enzymes were at least partly responsible for the resistance phenotype [7]. A study in 2013 of two sites near Kinshasa found that *An. coluzzii* were resistant to DDT and permethrin but fully susceptible to propoxur, bendiocarb and deltamethrin. Pre-exposure to PBO did not restore susceptibility in this population [8]. A recent study carried out from 2013 to 2016 found *An. gambiae* s.l. populations were resistant to permethrin in five of seven provinces studied in 2016 (Lodja and Kabondo located centrally, Mikalayi in the South, Kingasani in the West, and Kalemie in the East). A significant impact of PBO showed that metabolic resistance was involved in four of these sites. Resistance to deltamethrin was observed in Mikalayi and Kabondo, whilst resistance to DDT was observed in all six provinces where monitoring was carried out [5]. A study in Kinshasa in 2015, found that *An. gambiae* s.l. were resistant to DDT, four types of pyrethroid, dieldrin and bendiocarb, and that whilst P450 enzymes were involved, they were only partly responsible for the resistance observed [4].

Point mutations in the voltage gated sodium channel (VGSC), the target for pyrethroids, at the L1014 locus (L995 using *An. gambiae* codon nomenclature [9]) typically cause knock down resistance (*kdr*) to pyrethroids (and DDT), and are widespread in *An. gambiae* s.l. [10]. Two resistance alleles are found at this locus, resulting from the replacement of the wild type leucine allele with phenylalanine, L1014F [11], or alternatively serine, L1014S [12]. Both alleles can occur in the same population [7, 13] and the frequency of both alleles appears to be increasing [14–18].

In DRC the *Vgsc*-1014F mutation was found at four locations in *An. gambiae* s.s. specimens collected in 2009 with allele frequencies ranging from 0.13 to 0.95 [3]. The L1014F *kdr* mutation was also detected in *An. coluzzii* at frequencies of 0.33 and 0.38 in two study sites near Kinshasa [8]. The L1014F mutation was found in *An. gambiae* s.l. in all five provinces studied in 2014 with extremely variable frequencies ranging from near fixation to near absence [5]. The *Vgsc*-1014S mutation has only been detected in one location in the North East where it occurred at high frequencies and co-occurred with both the *Vgsc*-1014F mutation and the wild type allele [7]. A study in Kinshasa, 2015, found both L1014S and L1014F, whilst the wild type allele was almost entirely absent [4].

Detection of the *kdr* mutations, which act as useful partial resistance diagnostics for pyrethroid resistance [10], is important for monitoring the spread of resistance in areas were vector control is principally carried out using pyrethroid treated nets. A number of different assays exist for the detection of *kdr* including allele specific PCR (AS-PCR) [11, 12], Heated Oligonucleotide Ligation Assay (HOLA), [19] Sequence Specific Oligonucleotide Probe Enzyme-Linked ImmunoSorbent Assay (SSOP-ELISA) [20], PCR-Dot Blot [21], and the widely-used real-time TaqMan qPCR probe assay [22]. However, all of these methods are reliant on performing two assays in order to detect both the *Vgsc*-1014F and *Vgsc*-1014S mutations. With the spread of both mutations across Africa [9] there is a growing need for an assay that can detect both resistant alleles and the wild type allele in a single assay to aid interpretation, increase throughput, and reduce the cost per specimen. A newly-developed assay utilizing Locked Nucleic Acid (LNA) probes to simultaneously detect all three alleles whilst utilizing the same quantitative PCR platform used for TaqMan assays is presented. LNA probes incorporate modified RNA nucleotides that significantly increase target affinity and the melting temperature (Tm) of an allele-specific probe. This allows very short allele specific oligonucleotides to be produced that have a high difference in Tm between the target sequence and any mis-match sequence. Utilizing this technology, it was possible to design probes to detect all three alleles reliably in a single qPCR reaction and this assay was used to explore *kdr* frequencies in samples from Nord Ubangi, DRC.

## Methods

### Mosquito collections and bioassays

As part of baseline entomological monitoring for a LLIN distribution campaign in Nord Ubangi, Equateur province in March–April 2016, three rural collection sites (Pambwa, 3.937433, 20.7726; Fiwa, 4.318532, 20.77830; Bassa, 4.267017, 21.283383) were selected in the area surrounding the major town of Gbadolite, near the border with the Central African Republic (Additional file 1).

*Anopheles* larvae were collected from roadside breeding sites in the proximity of Fiwa and Pambwa, but none were detected around Bassa. Adult mosquito collections were carried out in all three villages using both manual and mechanical (‘Prokopack’) aspirators [23]. Blood-fed female *Anopheles* were maintained in a field insectary in Gbadolite until egg-laying. Larvae collected directly, and those raised from eggs, were reared until the adult stage and 3–5 day old females were used for insecticide susceptibility testing.

All mosquitoes were identified to species group using phenotypic keys [24] and insecticide testing was carried out on *An. gambiae* s.l. to assess the prevalence of resistance to permethrin (0.75%) and deltamethrin (0.05%) with and without PBO (4%) using standard World Health Organization (WHO) protocols [25]. A limited number of tests were also performed with DDT (4%) and the carbamate insecticide, bendiocarb (0.1%). Negative controls were carried for all insecticide assays. Mosquitoes were pre-exposed to PBO for one hour before exposure to the control paper in synergist assays. Abbotts correction was carried according to WHO criteria. All mosquitoes were stored on silica gel in 0.2 ml tubes for later DNA analyses.

### DNA extraction and molecular species identification

DNA was extracted from individual mosquitoes using Nexttec Biotechnologie GmbH extraction plates according to manufacturer’s instructions; or from legs using the following method. Two mosquito legs were removed and placed in 20 μl of 1 × STE buffer in a 0.2 ml PCR tube. Samples were incubated for 30 min at 95 °C. The supernatant was used as template for subsequent PCR reactions.

Mosquitoes from insecticide bioassays previously identified as *An. gambiae* s.l. were identified to species level by SINE [26], using 1 μl of DNA in a 25 μl reaction, with bands visualised on a 2% TAE agarose gel.

### TaqMan assays for resistance mutations

*Vgsc*-L1014F and *Vgsc*-L1014S mutations were screened using the TaqMan assay [22]. Results were analysed using AriaMX software V1.5 and the fluorescence (ΔR) threshold adjusted manually for each dye, if necessary, to enable the correct scoring of positive controls. Results for ΔR last, the final baseline-corrected fluorescence reading as measured in the last cycle, were then exported into Microsoft Excel for analysis and the genotype at locus 1014 determined by combining results from the two assays. Specimens were also screened for the presence of the *Vgsc*-N1575Y and *Ace*-*1*-G119S resistance mutations using TaqMan assays [27, 28].

### LNA-*kdr* assay for locus 1014

Primers were designed to amplify a single 141 bp region surrounding the *Vgsc*-1014 codon. LNA probes were designed according to suggested parameters [29]. The three probes were optimized utilizing the online IDT Biophysics software [30] to give an off-target Tm difference of at least 10 °C whilst keeping the exact match target Tm within 3 °C to allow target binding but preventing non-target binding of each probe within a single assay. All primers and probes were synthesised by IDT; sequence details are given in Table 1.

**Table 1:** Sequences of primers and probes used in the LNA kdr assay with 5′ and 3′ modifications indicated

Name	5′ Fluorescence modification	Sequence	3′ Quencher modification
VGSC-F		CGTGTGCTATGCGGAGAATG	
VGSC-R		CGATCTTGGTCCATGTTAATTTGC	
Kdr-Leu	HEX	A+CGA+C+T+AAAT+TTC+C	IBFQ
Kdr-Phe	6-FAM	A+CGA+C+A+AAAT+TTC+C	IBFQ
Kdr-Ser	Cy5	+CGA+C+T+GAAT+TTC+C	IBRQ

+, preceding a base indicates it is a LNA nucleotide

Each assay contained a final concentration of 1× PrimeTime Master Mix (IDT) or 1× Luna Universal qPCR Master Mix (NEB), 0.1 μM for each of the three probes (LNAkdr-Ser:Cy5, LNAkdr-Phe:Fam, LNAkdr-Leu:Hex), 0.2 μM of primers (VGSC-F, VGSC-R) in a total reaction volume of 10 μl with 1 μl of DNA template. Reactions were set up in optical PCR tubes and run on an AriaMX qPCR cycler with Fam, Hex and Cy5 filters. Reaction conditions were 95 °C for 3 min, followed by 40 cycles of 95 °C for 5 s and 60 °C for 30 s, giving a total run time of 52 min. Results were analysed using the AriaMX software V1.5 and the endpoint fluorescence (ΔR Last) threshold adjusted manually for each dye if necessary to enable the correct scoring of controls. Due to current software limitations automated analysis is restricted to bi-allelic genotype calling. Therefore, values for ΔR Last and genotype calling thresholds were exported to Microsoft Excel; ΔR Last values exceeding the threshold were counted as a positive call for each allele. The *kdr* genotype at locus 1014 was then determined for each sample. The R package Plotly [31] was used to produce a 3D scatterplot for all genotypes. To verify the method, final genotype calls were then compared to those produced in the TaqMan *kdr* assays from endpoint fluorescent data. To provide additional testing of the LNA-*kdr* assay for suitability across Africa, 172 *An. gambiae* s.s. samples, collected from four districts in Eastern Uganda in 2017 were also included.

Allele frequencies were calculated from the genotypes observed in the LNA assay. Association of genotype and susceptibility phenotype were analysed using Χ^2^ tests, and odds ratios were calculated using Poptools 3.2. The R package Genetics [32] was used to carry out an Exact test of Hardy–Weinberg Equilibrium.

## Results

### Species and phenotypic resistance in Nord Ubangi

Over 250 female mosquitoes were obtained from house collections in Fiwa, Pambwa and Bassa (Nord Ubangi), the majority of which (84%) were identified morphologically as *An. gambiae* s.l. *Anopheles funestus* was found in significant numbers (28%) from house collections in Pambwa. Larval collections from sites around Fiwa and Pambwa yielded only *An. gambiae* s.l.

Molecular species identification of *An. gambiae* s.l. showed that the predominant vector collected in all sampling areas was *An. gambiae* s.s. *Anopheles coluzzii* was found in larval collections from only one site (Fiwa) at low frequency, 12.5% (N = 4 of 32). This species was not detected in any other site.

**Fig. 1:**
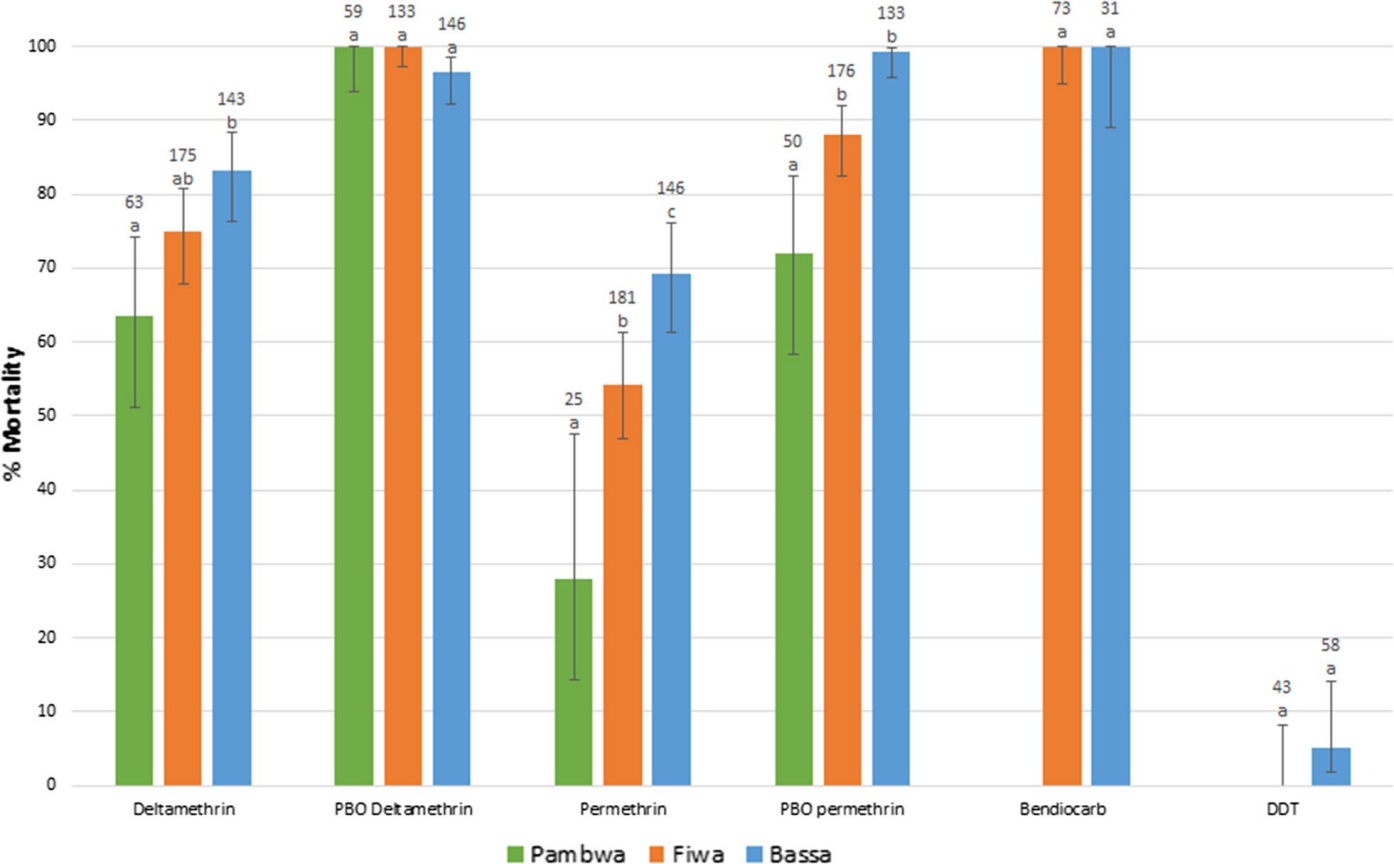
Percentage mortality to various insecticides with and without prior PBO exposure in each of the three study sites. Error bars show 95% binomial confidence intervals. The number of individuals tested is shown above each bar. For each insecticide, populations with significantly different mortalities (P < 0.05) are indicated by different letters whilst populations without significantly different mortalities share the same letter (z-ratio for independent proportions)

Adults reared from larval collections from Fiwa and Pambwa, and from F1 adults reared from eggs obtained from females in house collections from all three study sites, were exposed to permethrin and deltamethrin with and without prior PBO exposure, and the insecticides bendiocarb and DDT according to the WHO protocol [25]. Control assays yielded low mortality (mean = 3%) and results were corrected using Abbott’s formula when mortality in the control assays was between 5 and 20%. Prior exposure to PBO in the control assays also yielded low mortality (mean = 4%), and was no higher than controls without PBO (χ^2^ = 0.52, P = 0.47). Bioassay test results are shown in Fig. 1.

Resistance to permethrin and deltamethrin was detected in each study site although susceptibility was partially restored when PBO was used in conjunction with permethrin, and nearly completely restored when used in conjunction with deltamethrin. Despite the relatively small geographical scale (sites are < 100 km apart) significant variation in pyrethroid resistance was detected between the sample areas (Additional file 1). Data from Fiwa and Bassa study sites suggest the populations are fully susceptible to bendiocarb but show very high resistance to DDT.

### Molecular resistance diagnostics

**Fig. 2:**
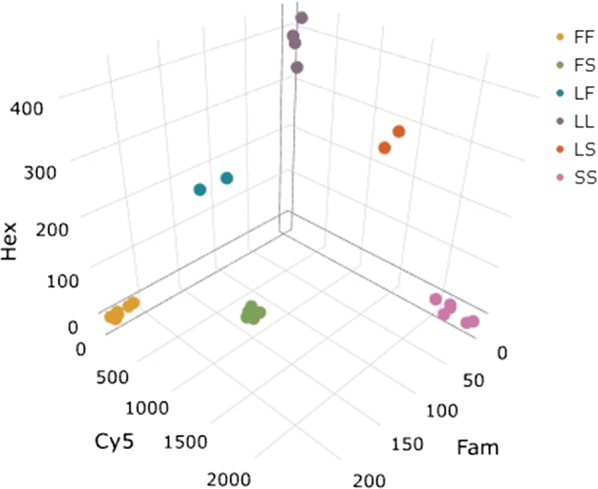
A 3D scatter plot showing distinct genotype groupings for *kdr* L1014 mutation as determined by the ΔR Last fluorescence of L1014L (Hex), L1014F (Fam) and L1014S (Cy5) probes in a single LNA-*kdr* assay

*Anopheles gambiae* s.s. control specimens of known genotype were screened using the newly-developed LNA *kdr* detection method. The assay gave distinct genotypes and clear cut-offs between wildtype, heterozygous and homozygous genotypes for all three alleles (Fig. 2). The assay was successful utilizing two different qPCR master mixes (IDT PrimeTime Master Mix or NEB Luna Universal qPCR Master Mix) suggesting other commercial qPCR mixes may also be suitable thus allowing both greater flexibility and potential for reductions in cost.

### LNA *kdr* detection versus TaqMan method

In total 356 *An. gambiae* from DRC and 172 specimens from Uganda (Additional file 2) were analysed for the *Vgsc*-1014 mutations using both the traditional TaqMan assays and the new LNA probe-based method in a blind assay. All samples analysed gave a result in both the TaqMan and LNA kdr assays. The LNA-*kdr* assay gave identical results to the TaqMan assays for all samples, which included homozygotes and heterozygotes for all three alleles. The Ugandan specimens provided a contrasting genotypic profile to those from DRC, with 1014S dominant, which allowed a more robust test of the LNA-*kdr* assay (Additional file 2).

The L1014F and L1014S mutations were discovered in *An. gambiae* s.s. specimens assayed from the three Nord Ubangi sample locations (Additional file 1) with the predominant genotype being *Vgsc*-1014F homozygotes in each area (Table 2). Wild type alleles were found only in two of the four *An. coluzzii* specimens collected from the village of Fiwa; both occurred as heterozygotes with the L1014F mutation. Population genotype frequencies conformed to Hardy–Weinberg expectations (Table 2). Association studies of genotype and susceptibility phenotype showed that the 1014F was significantly associated with survival to permethrin (χ^2^ = 10.43, P < 0.01; odds ratio = 3.7), deltamethrin (χ^2^ = 6.86, P < 0.05; odds ratio = 3.8) and with permethrin with prior PBO exposure (χ^2^ = 6.71, P < 0.05; odds ratio = 2.9). Note that in each case the tests involve comparison of to 1014S, rather than 1014L, which was absent.

**Table 2:** Genotype frequencies for the L1014 kdr mutation in *Anopheles gambiae* s.l. specimens from DRC. Tests of Hardy–Weinberg equilibrium (χ^2^) with corresponding P-values are shown

L1014 genotype	*Anopheles gambiae* s.s.			*Anopheles coluzzii*
	Bassa (N = 100)	Pambwa (N = 92)	Fiwa (N = 161)	Fiwa (N = 4)
FF	0.79	0.82	0.64	0.5
FS	0.20	0.17	0.29	0.00
SS	0.01	0.01	0.07	0.00
LF	0.00	0.00	0.00	0.5
Χ^2^	0.05	0.02	2.85	0.44
P value	1.00	1.00	0.10	1.00

The LNA-*kdr* assay was not subject to false positives resulting from non-specific binding of the mutant alleles to wild type probe as occurs in the TaqMan assays, resulting in more robust determination of genotype (Additional file 3). Non-specific binding can lead to a 1014F/S heterozygote sample being initially scored as having all three alleles meaning manual evaluation of the data and correction of the combined genotype is needed (Additional file 3). Importantly, in such cases, failure to carry out both Taqman-*kdr* assays may result in the reporting of false positives for the wild-type allele.

Molecular analysis of the third known *An. gambiae* resistance mutation N1575Y (N1570Y using *An. gambiae* codon numbering) in the voltage-gated sodium channel found the mutation was present in both *An. gambiae* s.s. and *An. coluzzii* populations albeit at very low frequency, 0.011 (N = 364) and 0.25 (N = 4) respectively. The N1575Y mutation was only found in conjunction with L1014F homozygote (Additional file 4). The important carbamate and organophosphate resistance mutation *Ace*-*1*-G119S was not found in any of the 368 specimens analysed.

## Discussion

The primary malaria vector species in the three study sites in Nord Ubangi was found to be *An. gambiae* s.s., although *An. coluzzii* was also found in low numbers. Data for this region of DRC are lacking, but previous studies in southern Equateur Province found only *An. gambiae* s.s. [1, 3]. The presence of *An. coluzzii* in Nord Ubangi further extends the known range of this species.

Molecular analysis revealed the *kdr* L1014F resistance mutation to be present at high frequency (over 0.79–0.90), and the *kdr* L1014S mutation found to be present at moderate frequency (0.1–0.21) across all three study sites (Table 2). The wild-type susceptible allele was not detected in *An. gambiae* s.s. but was found in the few *An. coluzzii* specimens analysed. The N1575Y mutation, also located in the VGSC, has a synergistic effect on pyrethroid and DDT resistance when combined with the L1014F mutation [27, 33]. Previously this mutation has been found in Burkina Faso, Ghana, Benin, Cameroon and Côte d’Ivoire [27, 34–36]. It has been detected in both *An. gambiae* s.s. and *An. coluzzii* [27, 34]. In this study the N1575Y was found at very low frequency in two of the three survey sites (Pambwa, 0.01 and Fiwa, 0.02) in *An. gambiae* s.s. The mutation was found at a frequency of 0.25 (N = 4) in *An. coluzzii* from Fiwa. This is first report of this mutation in DRC.

This study detected confirmed phenotypic resistance to deltamethrin, permethrin and DDT; the presence of both L1014 *kdr* resistance alleles at high frequency, the absence of the wild type L1014L mutation and the first detection of N1575Y. Such a resistance profile, mediated by both target site and metabolic mechanisms (indicated by PBO results) may provide challenges for LLIN-based vector control programmes in this region of DRC. However, susceptibility to bendiocarb and the absence of the Ace-1 G119S mutant suggest that indoor residual spraying (IRS) using non-pyrethroid formulations may not be compromised. Interestingly, the effect of PBO was not consistent across the two pyrethroids tested, with near-full susceptibility restored with deltamethrin, but not permethrin, suggesting a different balance in the contribution of different resistance mechanisms, with the latter perhaps more dependent upon target site resistance.

The LNA-kdr assay was found to perform as well as the TaqMan kdr assay in all populations of *An. gambiae* s.s. analysed, but with substantial time savings (67% reduction in run time) and since it utilizes non-proprietary probes also permits large cost savings (75%), which may be reduced further by use of different amplification master mixes. In addition, this single assay detection method may reduce the reporting of false positive wild-type alleles and permit the discovery of resistance alleles in places where they are not yet being screened for in single mutant detection assays. The development of a single assay for detection of both *kdr* alleles will allow rapid, reliable and low-cost screening of these important resistance mutations facilitating the monitoring of resistance across Africa.

## Conclusion

*Anopheles gambiae* s.s. populations in Nord Ubangi show insecticide resistance mediated by both metabolic mechanisms and target site mutations. The LNA-*kdr* assay developed was able to simultaneously detect all three *kdr* alleles in Nord Ubangi and has proved a reliable diagnostic tool for *An. gambiae* s.s. populations in which both 1014S and 1014F *kdr* alleles co-occur.

## Additional files

**Additional file 1.** Collection sites in the Gbadolite region of DRC. (A) Location of Gbadolite in DRC (shown in red) within Equateur province and the capital city, Kinshasa ★. (B) Fine-scale map of collection sites.

**Additional file 2.** Genotype frequencies for the VGSC L1014 mutations in *Anopheles gambiae* s.s. specimens from Uganda.

**Additional file 3.** Taqman genotype plots demonstrating mis-calling of genotype due non-specific binding of the mutant alleles. Mis-called genotypes are circled in black and the actual genotype indicated. A: Taqman kdr for L1014F detection. B: Taqman kdr for L1014S detection.

**Additional file 4.** Genotype frequencies for the VGSC N1575Y mutation *in Anopheles gambiae* s.l. specimens from DRC.
