# Supplementary figures and images for: Insecticide resistance in Anopheles gambiae from the northern Democratic Republic of Congo, with extreme knockdown resistance (kdr) mutation frequencies revealed by a new diagnostic assay
Source: Malar J. 2018 Nov 6;17:412. doi: 10.1186/s12936-018-2561-5 (PMC6219172; doi:10.1186/s12936-018-2561-5)

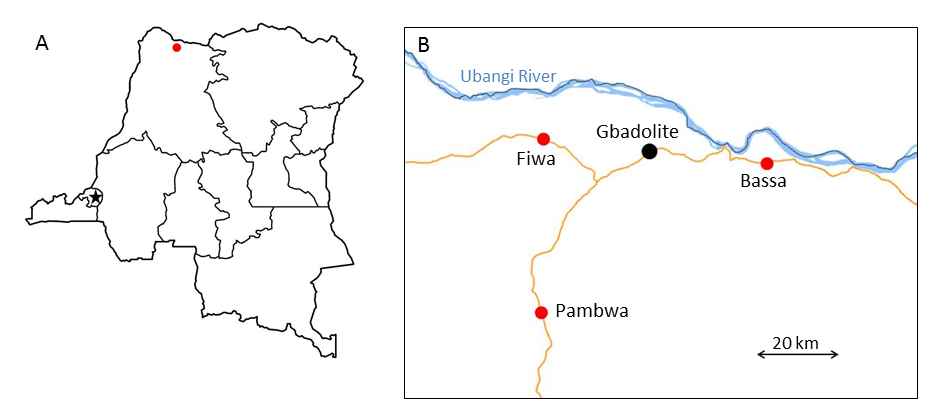

Supplement: Supplementary file 1 — Additional file 1. Collection sites in the Gbadolite region of DRC. (A) Location of Gbadolite in DRC (shown in red) within Equateur province and the capital city, Kinshasa ★. (B) Fine-scale map of collection sites. [file 12936_2018_2561_MOESM1_ESM.tif]

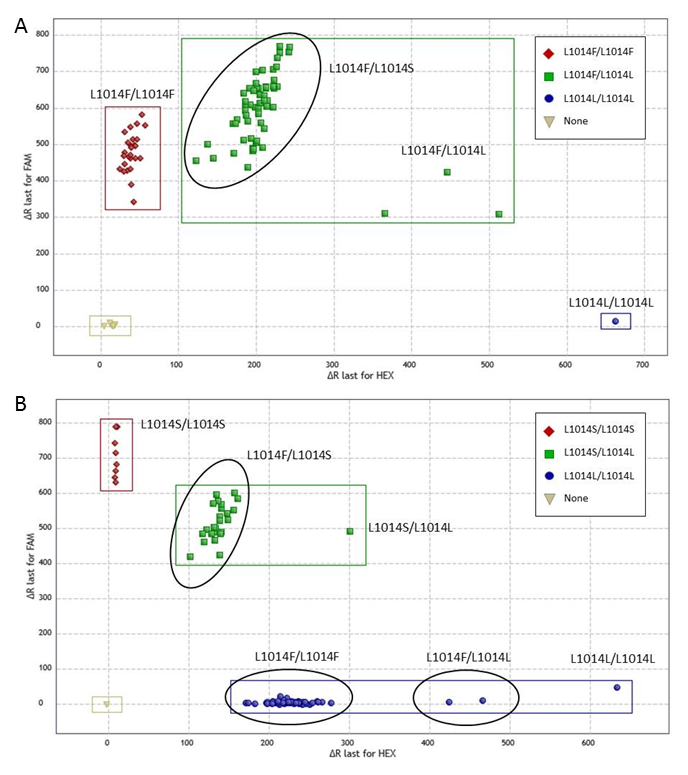

Supplement: Supplementary file 3 — Additional file 3. Taqman genotype plots demonstrating mis-calling of genotype due non-specific binding of the mutant alleles. Mis-called genotypes are circled in black and the actual genotype indicated. A: Taqman kdr for L1014F detection. B: Taqman kdr for L1014S detection. [file 12936_2018_2561_MOESM3_ESM.tif]
