# Supplementary material for: Insecticide resistance in Anopheles gambiae from the northern Democratic Republic of Congo, with extreme knockdown resistance (kdr) mutation frequencies revealed by a new diagnostic assay
Source: Malar J. 2018 Nov 6;17:412. doi: 10.1186/s12936-018-2561-5 (PMC6219172; doi:10.1186/s12936-018-2561-5)
Supplement: Supplementary file 2 — Additional file 2. Genotype frequencies for the VGSC L1014 mutations in Anopheles gambiae s.s. specimens from Uganda. [file 12936_2018_2561_MOESM2_ESM.docx]

| L1014 genotype | Frequency (N=172) |
| --- | --- |
| SS | 0.95 |
| FS | 0.04 |
| LS | 0.01 |
