# Supplementary material for: Insecticide resistance in Anopheles gambiae from the northern Democratic Republic of Congo, with extreme knockdown resistance (kdr) mutation frequencies revealed by a new diagnostic assay
Source: Malar J. 2018 Nov 6;17:412. doi: 10.1186/s12936-018-2561-5 (PMC6219172; doi:10.1186/s12936-018-2561-5)
Supplement: Supplementary file 4 — Additional file 4. Genotype frequencies for the VGSC N1575Y mutation in Anopheles gambiae s.l. specimens from DRC. [file 12936_2018_2561_MOESM4_ESM.docx]

|  | *Anopheles gambiae* s.s. | | | *An. coluzzii* |
| --- | --- | --- | --- | --- |
| 1575 frequency | Bassa  (N=104) | Pambwa  (N=96) | Fiwa  (N=164) | Fiwa  (N=4) |
| N | 1.00 | 0.99 | 0.98 | 0.75 |
| NY | 0.00 | 0.01 | 0.02 | 0.25 |
| YY | 0.00 | 0.00 | 0.00 | 0.00 |
